# Population genetic structure of *Hymenopellis radicata* germplasm resources based on genome re-sequencing

**DOI:** 10.3389/fmicb.2024.1287641

**Published:** 2024-01-24

**Authors:** Luping Cao, Delong Yang, Qin Zhang, Yanqing Ni, Wensheng Li, Rencai Feng, Wen Mu, Xu Zhao

**Affiliations:** ^1^Institute of Urban Agriculture, Chinese Academy of Agricultural Sciences, Chengdu, China; ^2^Chengdu National Agricultural Science and Technology Center, Chengdu, China; ^3^College of Life Science and Technology, Gansu Agricultural University, Lanzhou, China; ^4^College of Food and Biological Engineering, Chengdu University, Chengdu, China; ^5^College of Agronomy and Biotechnology, Southwest University, Chongqing, China

**Keywords:** *Hymenopellis radicata*, whole genome resequencing, SNP, INDEL, population genetic structure

## Abstract

Through whole-genome re-sequencing of 18 *Hymenopellis radicata* germplasm resources collected from diverse regions in China, we identified significant variations in the form of Single Nucleotide Polymorphisms (SNPs) and Insertions and Deletions (InDels). These variations were comprehensively annotated, shedding light on the mutation types present in the entire genome of the *H. radicata* germplasm. This analysis revealed the number and position information of each mutation and provided insights into the overall genomic landscape of *H. radicata* germplasm. Utilizing SNP data, we delved into the population structure of the 18 *H. radicata* germplasm resources. The results indicated the presence of 2,335,179 Indel sites and 12,050,448 SNP sites. The population structure analysis unveiled two distinct subgroups among the *H. radicata* germplasm resources. Phenotypic statistics, principal component analysis, and phylogenetic tree results echoed the findings of the population structure analysis. Different strains of *H. radicata* from various regions in China exhibited notable differences in genetic diversity, mycelial growth rate, yield, and fruiting body characteristics. Significant disparities were observed between the two subgroups, while strains within each subgroup shared common characteristics. This research establishes a solid foundation for integrating *H. radicata* into diverse breeding programs. The data underscore the potential of *H. radicata* for genetic improvement and exploitation in breeding initiatives, paving the way for future advancements in this field.

## 1 Introduction

The Physalacricea *Hymenopellis radicata* mushroom, commonly known as Changgengu, belongs to the Basidiomycota, Agaricales, and Physalacriceae families (Yang et al., 2009). As a delectable and nutritionally rich rare edible fungus, it predominantly thrives in Sichuan, Yunnan, and Guizhou provinces (Hou et al., 2022). The fruiting body of *H. radicata* contains active components such as polysaccharides (Wang et al., 2018; Pan et al., 2022) and flavonoids (Zhang et al., 2022), making it a valuable source of natural antioxidants and medicinal supplements for food, pharmaceuticals, and health products. Despite efforts in optimizing cultivation methods (Xiao et al., 2021) and extracting bioactive components (Zhang et al., 2021) from domesticated strains, the existing cultivated strains have low yields and inconsistent quality. The breeding of *H. radicata* has proven to be comparatively inefficient, compounded by issues of disorganized strain management in the market, leading to challenges such as the presence of the same species and name. The industry’s growth is intricately tied to the quality of *H. radicata* strains. Given its widespread presence in the wild across our country, we can effectively harness these excellent resources to enhance the genetic diversity of the strains, facilitating the breeding of high-yield, superior, and unique *H. radicata* strains in China.

In *H. radicata* cultivation, the fruiting body characteristics often exhibit high variability, with phenotypic traits closely linked to genotype and environment. The unstable phenotypic characteristics pose challenges to genetic improvement in *H. radicata*. The utilization of existing genomic resources and the generation of additional genomic data can help unravel the molecular basis of phenotypic variation. In recent years, molecular resources for *H. radicata* have expanded, including *de novo* transcriptome assembly (Zhu et al., 2023), ISSR labeling (Lian et al., 2023), and fingerprint construction (Gong et al., 2022). However, these studies primarily focused on genome structure analysis, strain identification, and classification, lacking a comprehensive discussion on the genetic diversity of *H. radicata* strains.

In the exploration of genetic diversity, resequencing technology is often employed, in addition to molecular markers, to detect genetic variation in samples. Whole-genome resequencing, a high-throughput sequencing technique, it is possible to sequence the genomes of various members of a species whose genome sequence is known and compare the variations between individuals or populations (Ley et al., 2008). Comparing whole-genome re-sequenced individuals with reference genome sequences allows for the discovery of numerous Single Nucleotide Polymorphisms (SNPs) and Insertions and Deletions (InDels) sites. A notable example is the analysis conducted by Shen et al. (2020), which explored the genetic diversity and population genetic structure of 28 strains of *Flammulina filiformis*. Another study by Shi et al. (2019) involved the preliminary resequencing investigation of 18 *Agaricus bisporus* strains, leading to the identification of the largest hybrid strains in the world through SNP, InDel, and SV detection.

This study undertakes the complete genome resequencing of 18 *H. radicata* strains to detect, filter, and subtype SNP and InDel locations. Group genetic structure analysis, utilizing SNP data, is employed to construct a systematic development tree and perform principal component analysis. Simultaneously, the study seeks the theoretical basis for the protection and utilization of *H. radicata* resources, genetic localization of important agricultural characteristics, and polymorphic breeding based on molecular markers. These objectives are pursued in conjunction with the physiological epithetic indicators of all strains. This research aims to contribute valuable insights to the genetic variation in edible fungi, paving the way for the development of new molecular markers to assist in breeding, aligning with the advancements brought about by resequencing technology in this domain.

## 2 Materials and methods

### 2.1 Source of the tested strains

The Institute of Urban Agriculture, Chinese Academy of Agricultural Sciences, supplied 18 specimens of *H. radicata* collected from diverse provinces and cities across China. Based on their respective origins, the 18 strains were categorized into three groups: A, B, and C. Strains in Group A originated from the eastern coastal area, those in Group B were sourced from central China, and Group C comprised strains from southwest China. Details regarding their names and locations are presented in Table 1.

**Table 1:** Breed number and origin of 18 *H. radicata* strains.

Number	Strains	Origin	Group	Number	Strains	Origin	Group
1	Or015	Fuzhou, Fujian	A	10	Or007	Linyi, Shandong	A
2	Or024	Fuzhou, Fujian	A	11	Or019	Biyang, Henan	B
3	Or022	Fuzhou, Fujian	A	12	Or020	Jiayu, Hubei	B
4	Or001	Zhangzhou, Fujian	A	13	Or017	Wuhan, Hubei	B
5	Or002	Zhangzhou, Fujian	A	14	Or023	Changsha, Hunan	B
6	Or004	Zhangzhou, Fujian	A	15	Or018	Xijiu, Guizhou	C
7	Or009	Gaoyou, Jiangsu	A	16	Or012	Chengdu, Sichuan	C
8	Or003	Jining, Shandong	A	17	Or013	Chengdu, Sichuan	C
9	Or034	Junan, Shandong	A	18	Or014	Chengdu, Sichuan	C

### 2.2 Strain culture and sample collection

Solid medium (Potato Dextrose Agar, PDA) was prepared using 1 L of distilled water, 200 g of potato, 20 g of glucose, and 15 g of agar. The mycelium and medium were separated using cellophane. The mycelium was cultivated on a complete plate for subsequent DNA extraction and sequencing. Specifically, it was cultured in a 9 cm diameter petri dish containing 20 mL of PDA.

### 2.3 DNA extraction and genome sequencing of *H. radicata*

DNA extraction was carried out using the CTAB method (Zhang et al., 2010) following the crushing of an appropriate amount of mycelial tissue with liquid nitrogen. The purity of the extracted DNA was assessed using a spectrophotometer (K5800, Kaiao, Beijing, China). DNA samples with distinct electrophoretic bands and 260/280 nm values falling between 1.6 and 1.8 were selected for further analysis. High-quality DNA samples meeting these criteria were sent to Wuhan Hope Group Biotechnology Co., Ltd. for sequencing on the Illumina platform. The DNA sample underwent subsequent processes, including fragmentation, purification, terminal repair, 3′ terminal adenylation, junction head, fragment screening, and fragment enrichment through PCR.

### 2.4 Sequencing quality control

Raw data obtained through sequencing is processed using base recognition to generate clean data. The initial sequencing data may contain adapter sequences and low-quality sequences. To ensure the quality of subsequent data analysis, the fastp (Chen et al., 2018) program is employed to enhance the quality of the original data. Data noise is then reduced through data filtering. The clean data undergoes rigorous filtering, resulting in high-quality data suitable for subsequent information analysis. This high-quality data is essential for generating reliable results in the subsequent information analysis.

The filtering steps include: (1) filtering Reads that contain the adapter (for paired-end sequencing, both ends of the Reads are removed if one end contains the adapter); (2) removing low-quality Reads (bases with quality values below Q15 in Reads constitute more than 40% of all bases; for paired-end sequencing, if one end is low-quality Reads, both ends of the Reads will be removed); (3) eliminating Reads with more than 5; and removing Reads with more than 1.

### 2.5 Compared with the reference genome

Following data filtering, the clean data underwent alignment with the reference genome utilizing the BWA-mem (Bolger et al., 2014) program, yielding alignment results in SAM format. Subsequently, the alignment findings were converted to BAM format and sorted according to chromosomal order using the SAMtools (Li et al., 2009) software. To ensure the quality of the data, duplicate reads were identified and removed using Picard software. After eliminating duplicates, the alignment results were scrutinized using Qualimap 2 (Okonechnikov et al., 2016).

### 2.6 Genomic variation detection and annotation

Based on the alignment results, the GATK (McKenna et al., 2010) software was employed to identify SNPs and InDels. Subsequently, the ANNOVAR (Wang et al., 2010) software was utilized to annotate the detected SNPs and InDels. The annotation results were then leveraged for subsequent analysis and interpretation. Finally, the outcomes were utilized to discern genetic variations and explore their potential biological roles.

### 2.7 Population analysis

Based on SNP data, the population structure of *H. radicata* was investigated through the ADMIXTURE (Alexander et al., 2009) program. The optimal subpopulation number, K, was determined via cross-validation error. The precise procedure involves studying the population structure across various K values. We employed 10 different seeds for 10 repetitions of the analysis. The outcomes were clustered using Pong (Behr et al., 2016) in 10 iterations, and the most representative results were chosen for visualization. For all samples, the IBS matrix was computed using PLINK (Purcell et al., 2007). Subsequently, a phylogenetic tree was constructed using the neighbor-joining algorithm in PHYLIP (Shimada and Nishida, 2017). Principal component analysis based on SNP divergence was then conducted using EIGENSTRAT (Price et al., 2006).

### 2.8 Statistics of biological characters of different strains

Firstly, activated strains were inoculated onto a solid PDA medium using a 5 mm punch. Subsequently, they were placed in a constant temperature incubator at 25°C to avoid light during cultivation. A “cross” method was employed to mark the starting line and date on the back of the plate with a pen when the mycelium made contact with the medium surface. As the mycelium reached approximately 1 cm from the dish edge, the ending line and date were marked. The distance between the starting and ending lines was measured to calculate the average growth rate of mycelium. Each strain had three parallels, and the average value was determined for comparison and analysis. The formula for calculating the average growth rate (V, mm/d) is: V = D/d.

Secondly, mushroom cultivation was conducted in Pengzhou, Sichuan province, using the soil covering method in small areas. Each strain was set up in three identical replicate areas, each comprising 15 bags. The mature bag cultivation method was employed in production, involving substrate mixing, bagging, inoculation, and mushroom development management. In each small area, 20 harvested fruiting bodies were collected for quantitative trait statistics. The quantitative trait testing method followed the Guidelines for the conduct of tests for distinctness, uniformity, and stability rooting shank mushroom [*Oudemansiella raphanipes* (Berk.) Pegler & T.W.K. Young] (National Plant New Variety Test Standardization Technical Committee, 2020), with specific methods outlined in Table 2. Fresh fruiting bodies were harvested daily when the mushroom caps were fully expanded but not yet curled upwards. The weight of the fruiting bodies was recorded for each small area, and the total yield for the entire cultivation period was calculated to determine the yield per bag.

**Table 2:** Methods for determining quantitative traits of fruiting bodies.

Number	Characters	Method
No. 1	Pileus diameter	Measure the length of number 1 in Figure 1, 20 replicates
No. 2	Pileus thickness	Measure the length of number 2 in Figure 1, 20 replicates
No. 3	Stipe length	Measure the length of number 3 in Figure 1, 20 replicates
No. 4	Stipe diameter	Measure the length of number 4 in Figure 1, 20 replicates

**Figure 1:**
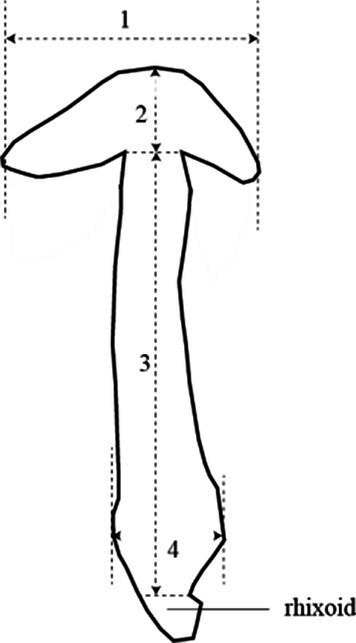
Schematic diagram of various parts of the fruiting body.

## 3 Results and analysis

### 3.1 Genome sequencing analysis

In this investigation, 18 strains of *H. radicata* were sequenced utilizing the Illumina platform (Table 3). Raw reads ranged from 142,018,200 M to 32,595,990 M. After filtering, the reads were refined to a range of 325,866,758 M to 14,196,060 M. Clean databases range in size from 4.89 GB to 2.13 GB, while raw databases also fall within the 4.89 GB to 2.13 GB range. The sequencing results met the criteria for re-sequencing data analysis because the clean data Q20 ranged from 93.26% to 98.64%, the clean data Q30 ranged from 85.33% to 96.09%, and the GC content ranged from 45.59% to 48.50%.

**Table 3:** Quality of re-sequencing data for 18 strains of *H. radicata.*

Sample	Raw reads number (M)	Raw data bases (G)	Clean reads number (M)	Clean data bases (G)	Clean data Q20	Clean data Q30	Clean data GC%
Or001	292,246,120	4.39	290,622,120	4.35	97.7930	93.5679	47.9731
Or002	276,714,300	4.15	274,909,180	4.12	97.9459	93.9989	48.1457
Or003	305,329,340	4.58	303,543,200	4.55	97.6902	93.3225	48.3862
Or004	284,761,740	4.27	283,114,880	4.24	97.6404	93.1906	48.0073
Or007	289,265,420	4.34	287,622,600	4.31	97.8011	93.5098	48.3826
Or009	286,800,600	4.30	285,116,860	4.27	97.5997	93.1156	48.5011
Or012	323,283,200	4.85	323,283,200	4.84	98.1250	94.6436	46.7751
Or013	325,831,840	4.89	325,831,840	4.88	97.3662	92.4603	46.5693
Or014	325,203,040	4.88	325,203,040	4.87	97.7535	93.5750	47.2989
Or015	325,959,900	4.89	325,959,900	4.89	98.3398	95.1298	46.6987
Or017	310,126,920	4.65	310,126,920	4.65	98.3387	95.1136	46.6430
Or018	309,087,860	4.64	309,087,860	4.63	98.4040	95.3102	46.7836
Or019	308,710,460	4.63	308,710,460	4.63	98.6416	96.0939	46.7383
Or020	309,491,240	4.64	309,491,240	4.64	98.1612	94.6065	46.3452
Or022	308,801,140	4.63	308,802,140	4.63	97.2164	92.0595	46.3672
Or023	309,706,700	4.65	309,706,700	4.64	98.2581	94.8948	46.7202
Or024	309,290,740	4.64	309,290,740	4.64	98.4996	95.5830	46.6569
Or034	142,018,200	2.13	142,018,200	2.13	93.2582	85.3306	45.5942

*H. radicata* IJFM A160 (GenBank accession: GCA_015501595.1) was used the reference genome for this study. Table 4 presents a comparison of the resequencing results from 18 *H. radicata* samples with the reference genome. More than 57.62% of the entire dataset successfully aligned to the reference genome in a double-ended manner, while over 32.90% of the total data showed precise matches with the reference genome. Moreover, over 67.21% of the total data were mapped to the reference genome. These findings highlight discrepancies between the experimental material and the reference genome, suggesting the need for further investigation to accurately characterize the genetic differences among these 18 samples. Such an analysis could offer valuable insights into the genetic variability of *H. radicata*.

**Table 4:** Statistics of genome alignment and coverage.

Sample	Total reads	Total mapped	Total pair reads	Total pair mapped	Properly paired	1X (%)	10X (%)
Or001	43,523,934	72.64%	25,479,978	58.54%	41.43%	54.32	42.04
Or002	40,743,576	72.50%	24,054,682	59.04%	41.54%	54.40	41.64
Or003	45,288,738	73.60%	26,094,136	57.62%	42.20%	54.31	42.33
Or004	41,780,177	71.56%	24,588,954	58.85%	39.86%	54.57	41.50
Or007	42,900,214	73.48%	31,521,273	73.48%	32.90%	54.11	41.76
Or009	43,575,939	74.16%	25,250,443	57.95%	42.89%	54.84	42.52
Or012	49,810,653	71.64%	31,494,048	63.23%	45.06%	54.72	43.34
Or013	50,909,618	71.48%	31,588,549	62.05%	42.83%	55.27	43.78
Or014	50,939,786	72.84%	31,552,918	61.94%	44.94%	55.17	43.90
Or015	50,193,035	71.87%	31,569,266	62.90%	44.25%	54.56	43.53
Or017	48,622,702	71.98%	30,072,205	61.85%	43.11%	55.16	43.65
Or018	48,138,851	72.01%	30,038,907	62.40%	44.00%	55.00	43.49
Or019	47,755,567	71.81%	30,009,770	62.84%	44.57%	54.65	43.07
Or020	48,798,468	70.84%	29,955,844	61.39%	40.89%	55.05	42.99
Or022	47,053,933	69.91%	29,973,347	63.70%	41.77%	54.62	42.90
Or023	48,393,451	71.93%	30,054,633	62.10%	43.32%	55.14	43.64
Or024	48,056,660	71.77%	30,055,329	62.54%	43.84%	54.94	43.39
Or034	20,877,936	67.21%	13,943,666	66.79%	40.34%	52.09	32.89

### 3.2 SNP and InDel sites of the strain of *H. radicata*

**Figure 2:**
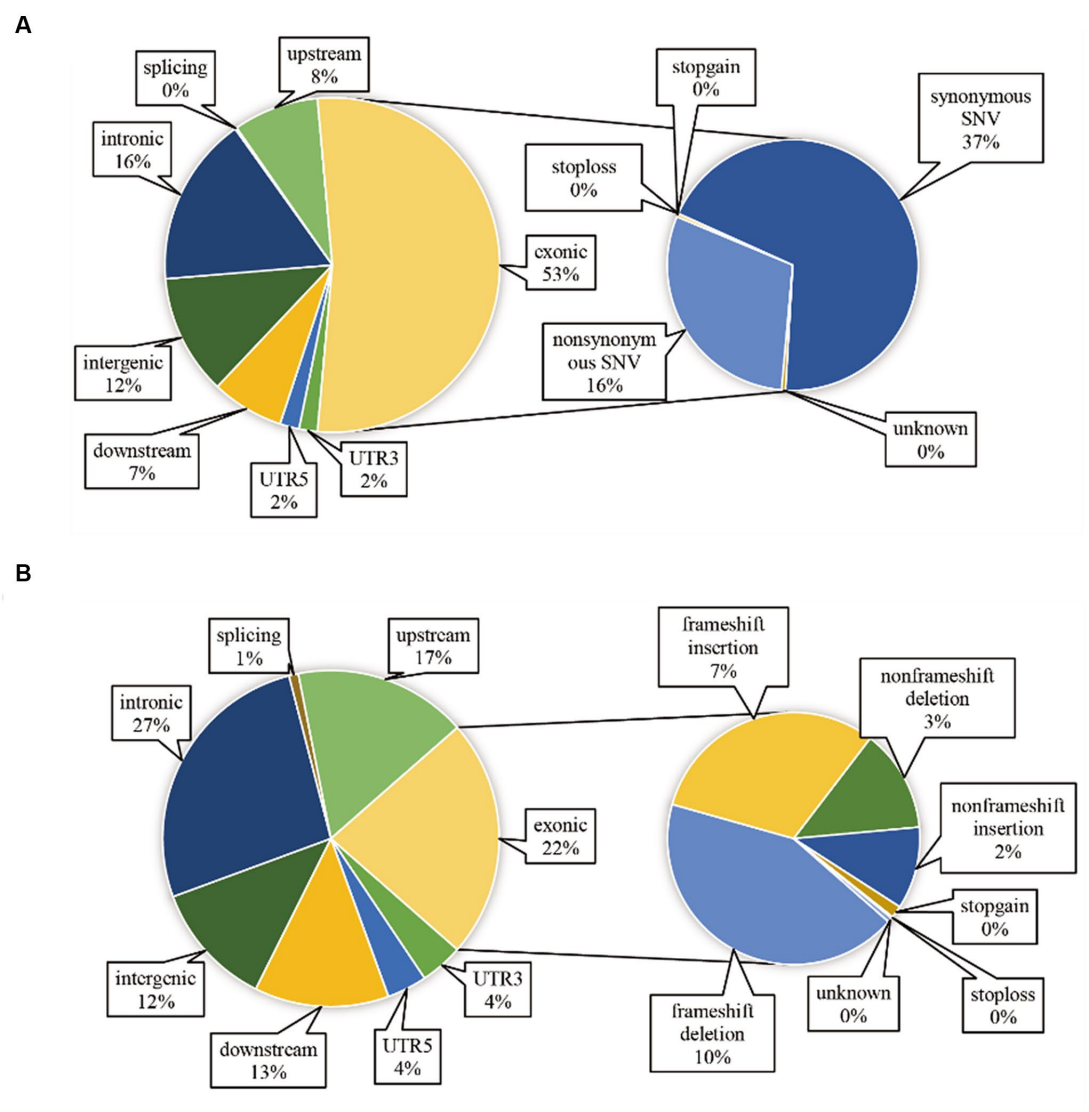
Localization and functional annotation of genetic variations in *H. radicata* strains. SNP functional annotation **(A)**; InDel functional annotation **(B)**.

After aligning with the reference genome, we identified 12,050,448 SNP sites and 2,335,179 InDel sites (refer to Table 5). Strain Or020 exhibited the fewest SNPs (502,973), while strain Or024 presented the highest count (692,787). The transitions ranged between 359,326 and 488,733, and transversions were observed between 143,627 and 204,054. InDel sites varied from the lowest in strain Or020 (92,542) to the highest in strain Or024 (136,963). Insertion sites ranged from 43,041 to 63,571, and deletion sites were observed between 49,501 and 73,392. Additionally, ANNOVAR software annotated the identified SNPs and InDels, and the annotations are displayed in Figure 2. Among the 18 *H. radicata* strains, SNPs located in the exon region constituted 53% of all identified SNPs. Among these, 70% were synonymous mutations, and 30% were non-synonymous mutations (Figure 2A). Regarding InDels, 22% of the total were located in the exon region, 27% in the intronic region, and 12% in the intergenic region (Figure 2B).

**Table 5:** SNP and InDel variation of 18 strains of *H. radicata* in comparison with the reference genome.

Sample	SNP			InDel		
	Transition	Transversion	Total	Deletion	Insertion	Total
Or001	477,282	198,026	675,308	68,233	60,511	128,744
Or002	480,300	198,969	679,269	68,640	60,319	128,959
Or003	478,923	199,418	678,341	69,966	61,529	131,495
Or004	446,140	182,689	628,829	62,148	54,803	116,951
Or007	479,231	199,379	678,610	69,794	61,274	131,068
Or009	485,903	202,872	688,775	72,221	62,693	134,914
Or012	487,966	203,843	691,809	73,192	63,186	136,378
Or013	487,839	203,783	691,622	73,065	63,322	136,387
Or014	488,423	203,966	692,389	73,361	63,598	136,959
Or015	479,016	199,415	678,431	70,238	61,552	131,790
Or017	488,053	203,822	691,875	73,013	63,241	136,254
Or018	488,383	204,034	692,417	73,325	63,559	136,884
Or019	488,012	203,803	691,815	73,156	63,357	136,513
Or020	359,326	143,627	502,953	49,501	43,041	92,542
Or022	479,768	199,655	679,423	70,064	61,484	131,548
Or023	487,935	203,731	691,666	73,117	63,434	136,551
Or024	488,733	204,054	692,787	73,392	63,571	136,963
Or034	441,988	182,141	624,129	60,797	53,482	114,279

### 3.3 Analysis of the population genetic structure of *H. radicata*

**Figure 3:**
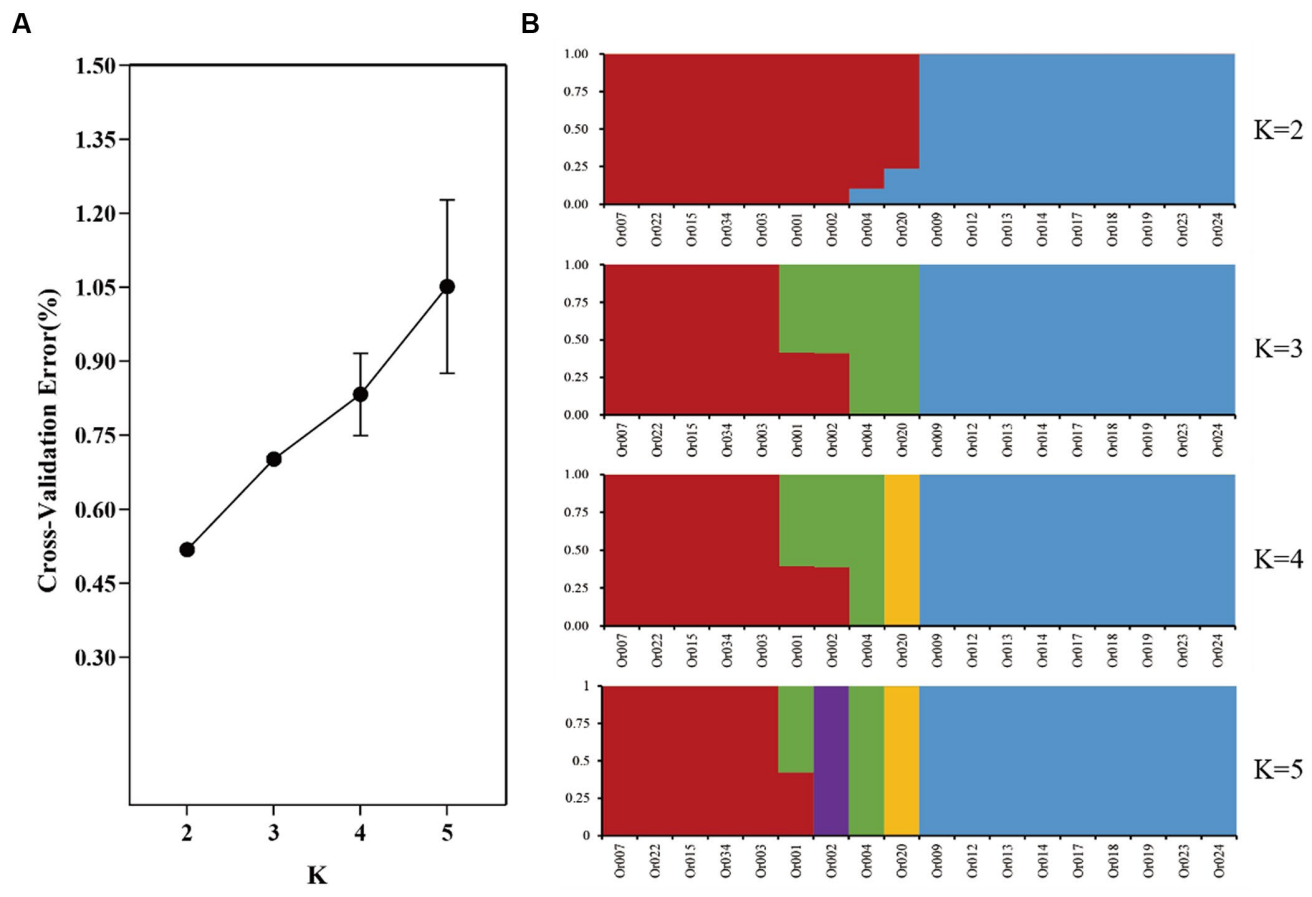
Cross-validation error rates **(A)** and the genetic structure of the *H. radicata* population **(B)** simulated by different K values.

PLINK 1.9 was employed to filter SNPs across the entire genome using the parameter “-Indep-pairwise 50 5 0.5”, based on the outcomes of the preceding investigation. For population stratification, 176,640 SNPs with loose linkages were utilized. The population structure was examined using ADMIXTURE version 1.3.0 for K values ranging from 2 to 5, and the ideal K value was determined through cross-validation error (CV). It is important to note that the model has higher reliability when the error rate at K = 2 is low, indicating that the most appropriate number of subgroups is two. Additionally, Figure 3A displays the result of the simulation analysis at different K values. At K = 2, the 18 germplasms were divided into two subgroups (red and blue), with strains Or004 and Or020 showing different levels of mixing between these two subgroups. This is speculated to be the result of genetic drift. When K = 3, strains Or004 and Or020 were categorized into a distinct subgroup, while strains Or001 and Or002 exhibited genetic elements from both subgroups, marking the beginning of further divergence between the two groups. At K = 4, strain Or020 branched off into its own subset, and at K = 5, strain Or002 also formed a new subgroup. The stable and genetically similar breeds were generally divided into two subgroups. Strains Or001, Or002, Or004, and Or020 are examples of strains that possess genetic components from two or more subgroups. As the number of subgroups increased, the phenomenon of genetic drift became more pronounced.

### 3.4 Comparison of mycelial growth rate between two subgroups of strains

**Figure 4:**
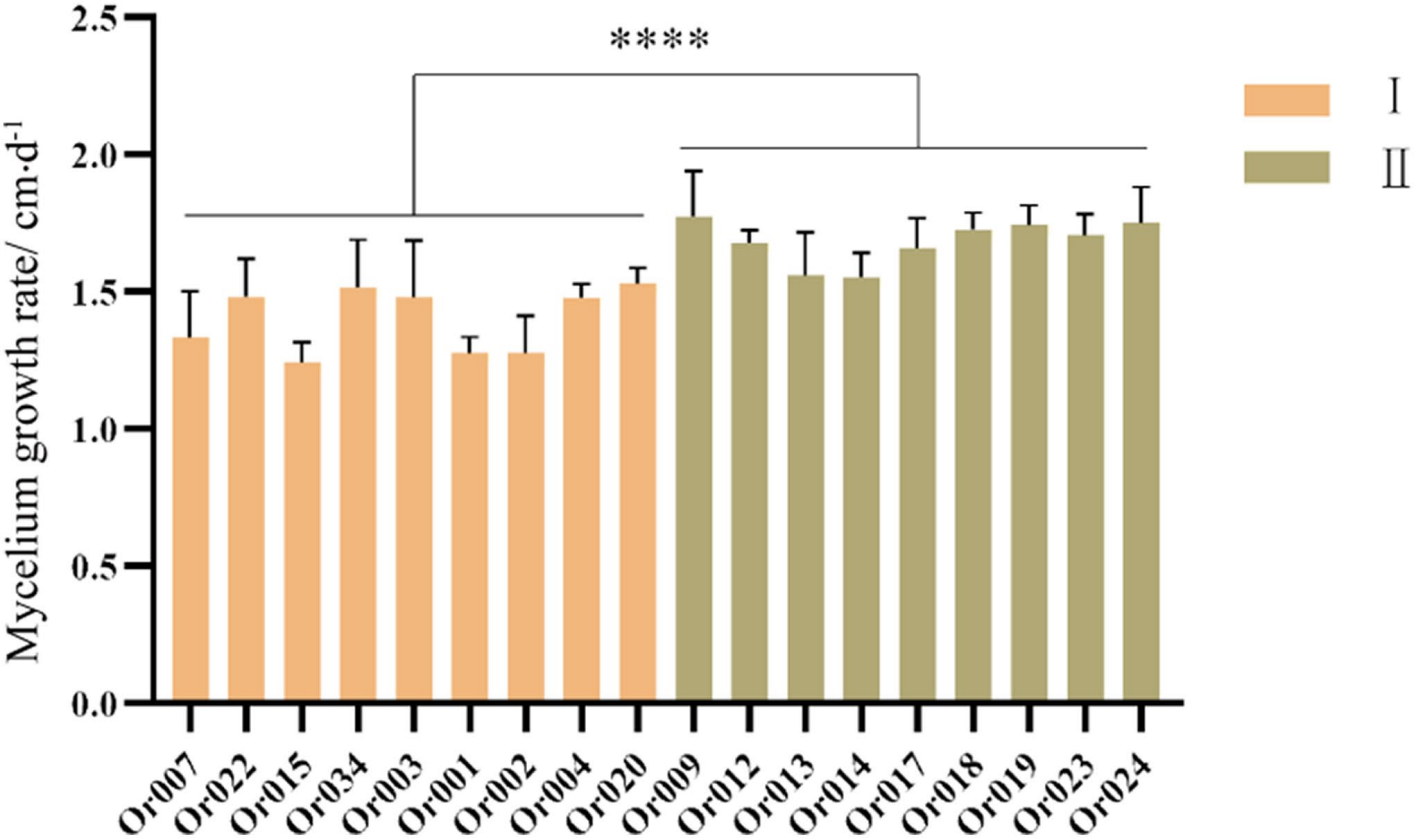
Comparison of mycelium growth rate among different strains of *H. radicata.* The value bar with **** indicates *p* < 0.0001.

The mycelial growth rate of strains in subgroup I ranged from 1.23 ± 0.074 cm/d to 1.53 ± 0.172 cm/d, while that of strains in subgroup II varied from 1.55 ± 0.086 cm/d to 1.77 ± 0.165 cm/d. The specific mycelial growth rates of each strain are shown in Figure 4. Statistical analysis revealed that strains in subgroup I grew significantly slower than those in subgroup II (*p* < 0.0001). This indicates that under identical culture conditions, strains in subgroup II have a significant advantage in terms of the mycelial growth rate index.

### 3.5 Comparison of single bag yield of two subgroups of strains

**Figure 5:**
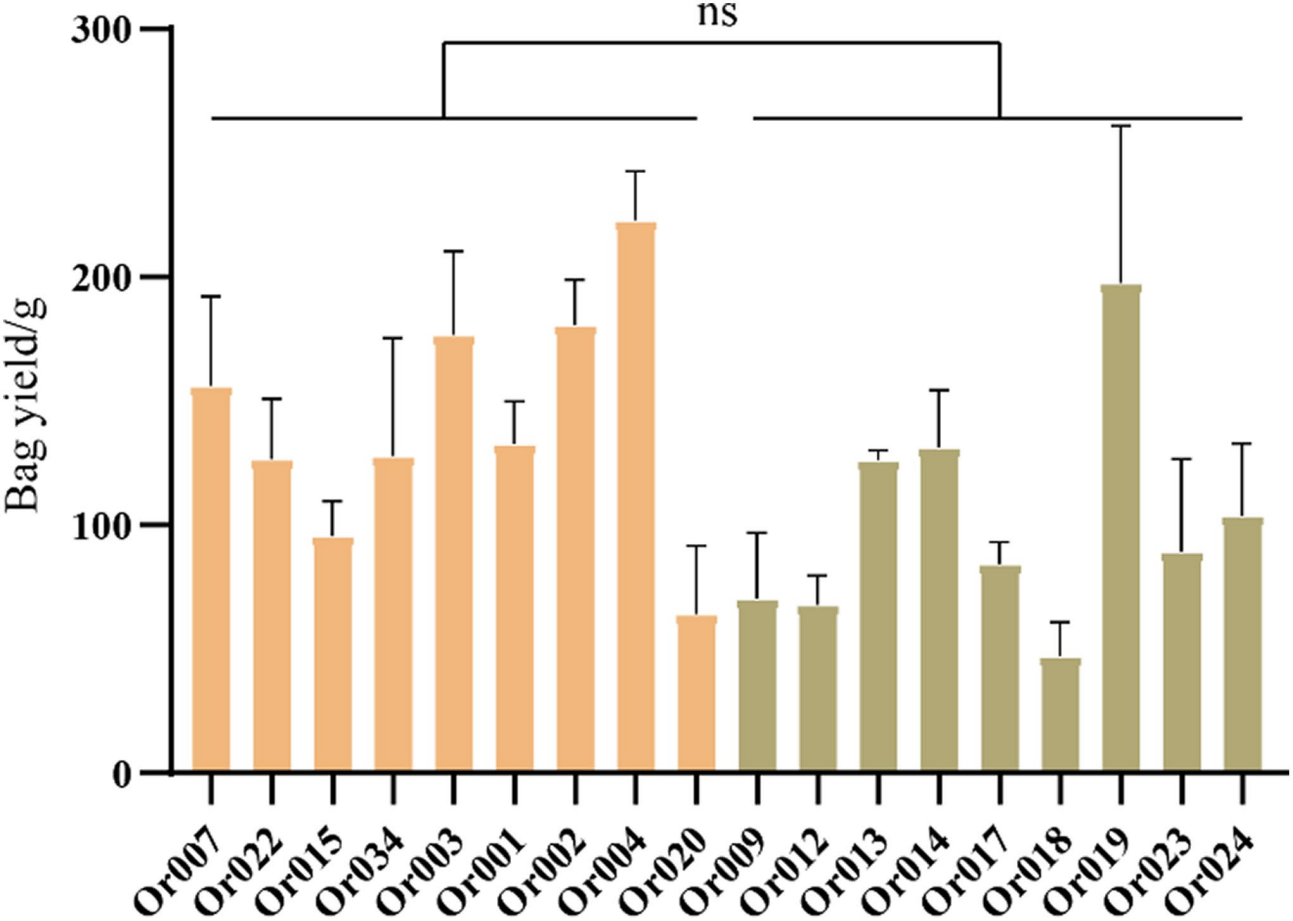
Comparison of yield between two subgroups of *H. radicata.* The symbol “ns” indicates not significant.

We collected yield data for all strains throughout their growth period and calculated the yield per bag, as shown in Figure 5. The yield of strains in subgroup I ranged from 64.10 ± 22.513 g/bag to 222.9 ± 13.930 g/bag, while the yield of strains in subgroup II varied from 47.08 ± 11.267 g/bag to 197.55 ± 51.631 g/bag. Although the average yield of strains in subgroup I was higher than in subgroup II, both subgroups contained strains with both high and low yields. Overall, the yield of strains within the two subgroups varied widely, showing no significant differences between them (*p* = 0.0593).

### 3.6 Comparison of the quantity and characteristics between two subgroups of strains

**Figure 6:**
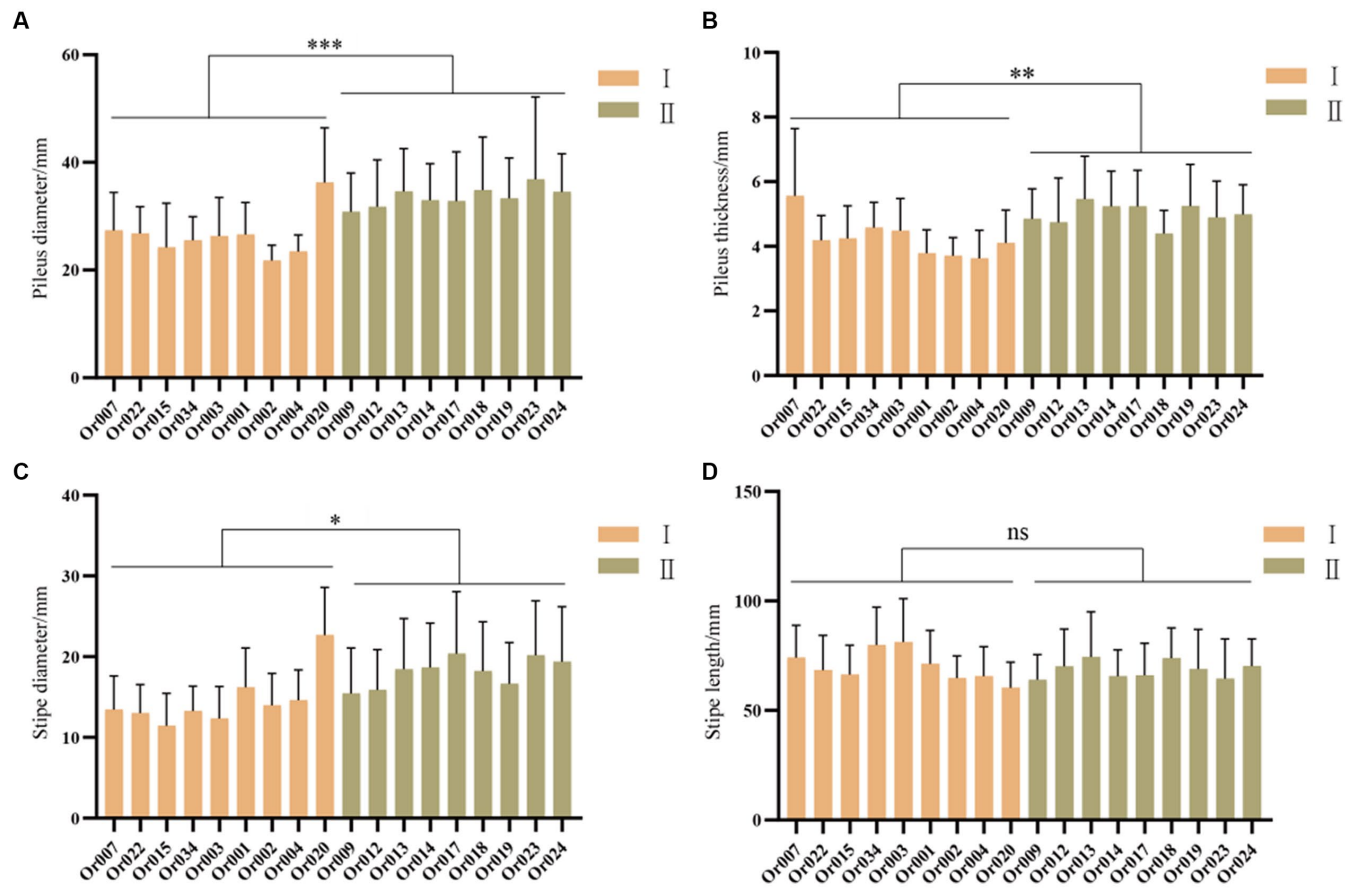
Comparison of fruiting body characteristics between two subgroups of *H. radicata.* **(A)** comparison of pileus diameter (*p* < 0.001); **(B)** comparison of pileus thickness (*p* < 0.01); **(C)** comparison of stipe diameter (*p* < 0.05); **(D)** comparison of stipe length (*p* = 0.4174). The symbol “ns” indicates not significant, the symbol “*” indicates *p* < 0.05; the symbol “**” indicates *p* < 0.01; the symbol “***” indicates *p* < 0.001.

As shown in Figure 6, the pileus diameter, pileus thickness, stipe diameter, and stipe length were measured for each strain. The strains from subgroup II had significantly larger pileus diameters (*p* < 0.001), pileus thickness (*p* < 0.01), and stipe diameter (*p* < 0.05) compared to those from subgroup I. However, there was no significant difference in stipe length between the two subgroups. On the whole, the strains in subgroup II exhibited larger pileus diameters, thicker pilei, and larger stipe diameters than those in subgroup I.

### 3.7 Population principal component analysis of *H. radicata*

**Figure 7:**
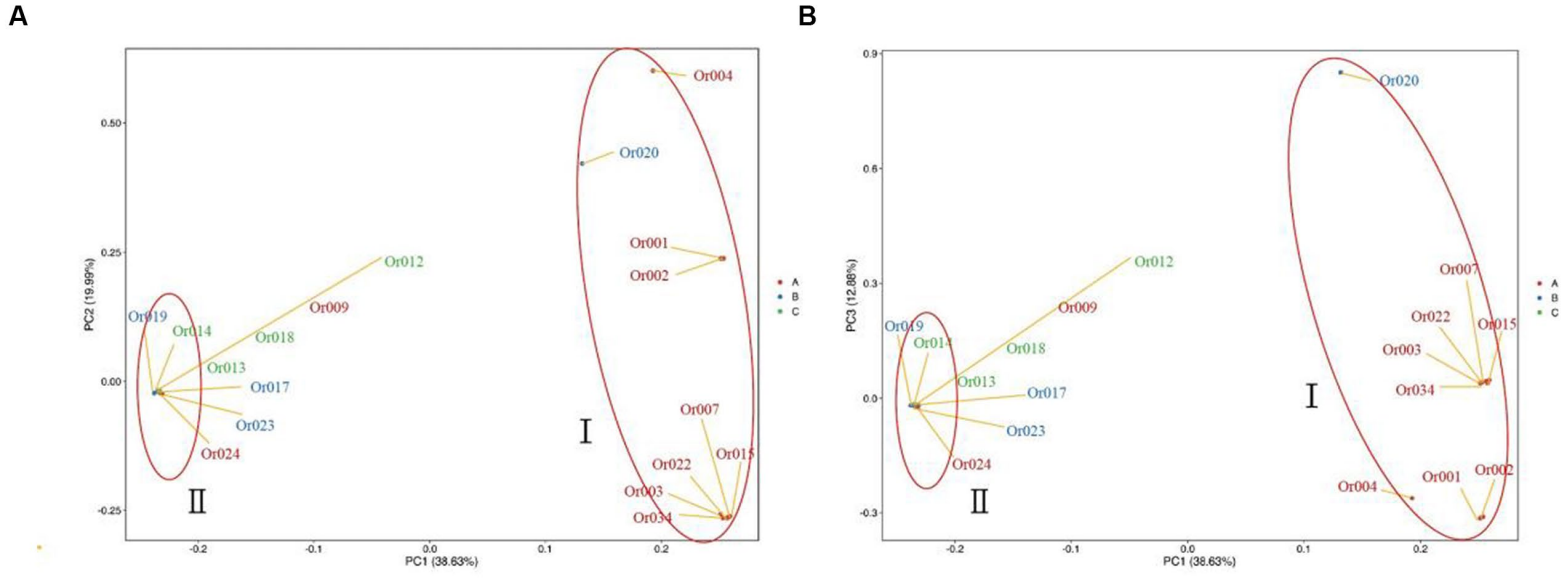
Principal component analysis (PCA) of 18 *H. radicata* strains. The numerical colors in the figure represent the strain names, and identical colors indicate similar geographical locations of the strain sources.

**Figure 8:**
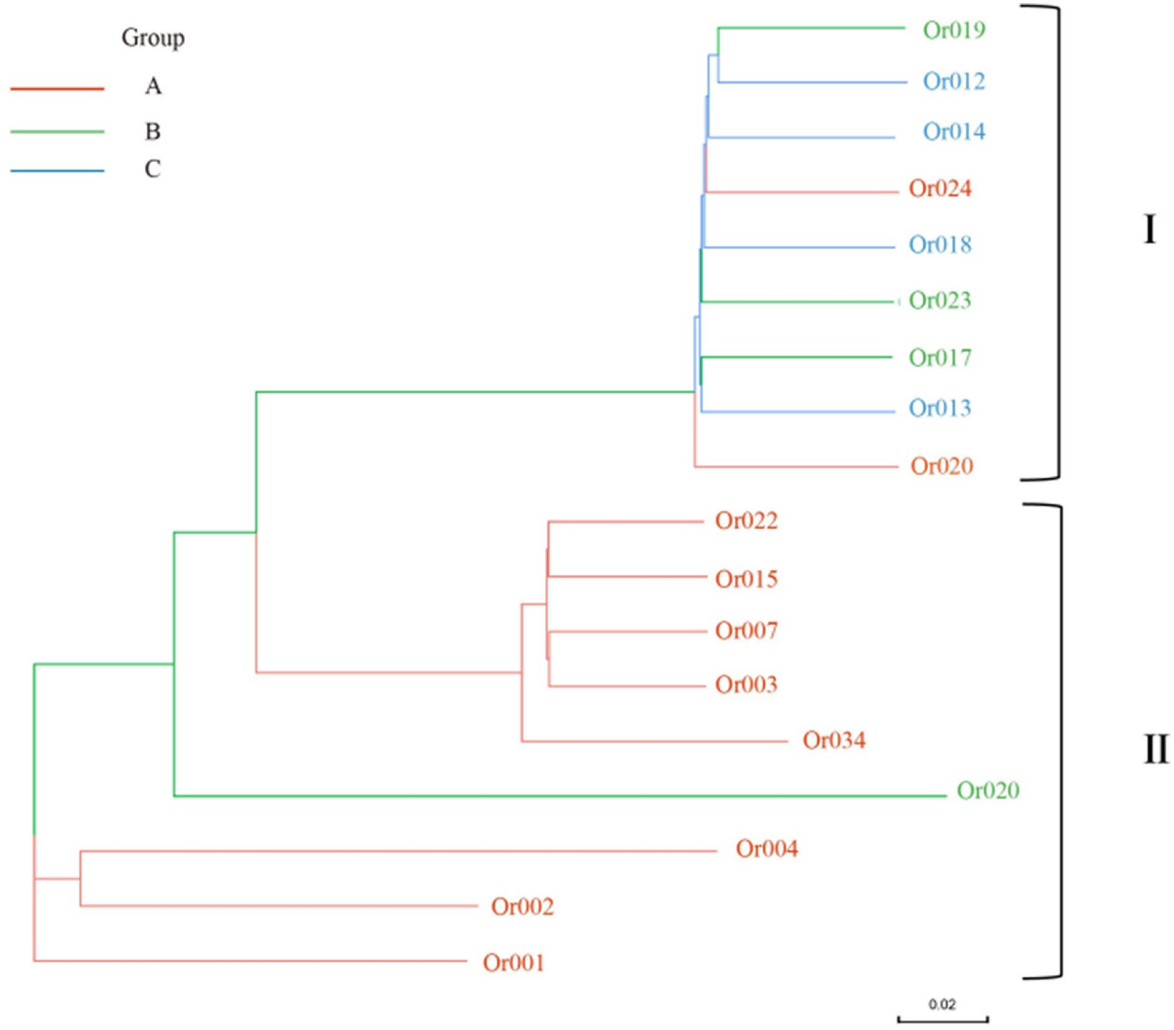
Phylogenetic tree of *H. radicata* based on SNP data. Red line A represents strains from Fujian, Jiangsu, and Shandong provinces; Green line B includes strains from Henan, Hubei, and Hunan provinces; blue line C comprises strains from Guizhou and Sichuan provinces.

Based on the extent of SNP differences detected in individual genomes, individuals were clustered into different subgroups and groups according to principal components, as depicted in Figure 7. Each point in the figure represents a sample, with greater distances between two points indicating more significant differences in the genetic backgrounds of the corresponding samples. The categorization of strains into two distinct groups, as determined by their locations and distances on the two-dimensional graph, aligns well with the results of the population genetic structure analysis (see Figure 8).

### 3.8 Cluster analysis of strain resources of *H. radicata*

The SNP identification data were used to construct a phylogenetic tree, which aided in understanding the evolutionary relationships among *H. radicata* populations from various geographical locations. According to the data, the strains in the two subgroups originated from four main branches. The first significant branch includes Or009, Or012, Or013, Or014, Or017, Or018, Or019, Or023, and Or024, which are present in Sichuan, Henan, Jiangsu, Hubei, Hunan, and Guizhou provinces. The second major branch comprises five strains: Or007, Or022, Or015, Or034, and Or003, found in Shandong and Fujian provinces. The third branch consists of strains Or001, Or002, and Or004, all located in Zhangzhou, Fujian Province. The unique strain Or020 originates from Jiayu, Hubei Province. As observed, strains conserved within the same region are closely related. There is minimal genetic difference between the strain populations from Shandong Province and Fujian Province. Strains from Hunan Province and Guizhou Province, as well as those from adjacent provinces, are genetically closer to those from Sichuan Province. Overall, the genetic analysis reveals a close relationship between the strains and their geographic distribution.

## 4 Discussion

We re-sequenced strains from various regions and successfully obtained a minimum of 2.3GB of clear data, with a Q30 exceeding 85.33% for each strain. The average genome coverage reached 71.93%. All strains were identified with a reference genome ratio of 12,050,448 SNP sites and 2,335,179 InDel sites. Using the SNP data for group genetic structure, principal component analysis, and constructing a systematic evolutionary tree, 18 strains were categorized into two subgroups. Furthermore, a statistical analysis of the growth rate, yield and fruiting body characteristics of the germ strains in the two subgroups revealed similarities within each subgroup and significant differences between them. The acquisition of this data contributes to understanding the genetic evolution of *H. radicata*.

The study of the evolution of edible fungi is a burgeoning topic. Numerous variables influence the diversification of mushroom populations, including geographical location. Several studies indicate that resequencing technology significantly enhance the effectiveness and accuracy of analysis by exploring the genetic diversity of germplasm resources of edible fungi in different regions (Bao and Xie, 2020). Branco et al. (2017) re-sequenced 55 strains of *Suillus brevipes* to analyze the differentiation process of these strains from different parts of the United States. They found that temperature was related to the genetic evolution of these strains, and some genes responsive to low-temperature stress were also discovered. Re-sequencing 33 *Pleurotus eryngii* populations from Europe and the Gobi Desert environment in China, Dai et al. (2024) discovered several genes involved in stress response and DNA repair connected to the adaptation to the Gobi Desert environment. Re-sequencing *Agaricus bisporus* strains from the Tibetan Plateau and the United States revealed significant independent divergence, as reported by Sun et al. (2019). Additionally, scientists identified a few crucial genes related to the cell wall and membrane that may play a role in the mushroom’s growth and development as well as its defensive mechanism against a hostile environment. The genes identified in the study employed to enhance the mushroom’s ability to adapt and survive in extreme environments. Moreover, the genetic diversity of the strains was analyzed at the later stages of mushroom molecular breeding. Zhu et al. (2019) re-sequenced *Ganoderma lucidum* isolates from South Korea and China and found many altered genes. The cultivation practices of mushrooms also impacted mushroom populations differently. Wild and domesticated Chinese *Lentinula edodes* were re-sequenced by Xiao et al. (2016). They separated the strains into three subgroups and filtered out 18 genes causing population variations, some of which were connected to stress responses. Re-sequencing 26 strains of *Sarcomyxa edulis* by Tian (2019). The results showed that the genetic distance between wild strain T24 and many cultivated strains was close, and there was gene exchange. It is speculated that these cultivated strains may have evolved from T24 strains. Shen et al. (2020) re-sequenced 28 wild and domesticated strains of *Flammulina filiformis* into five subgroups that were compatible with the breeding history of *F. filiformis*. Resequencing offers the benefit of obtaining information on variant genes as well as variable loci, crucial for environmental adaptation and population divergence. This has the potential to provide insight into the development of new varieties and offer new genetic resources for future breeding. It is also useful for understanding species genetic diversity.

### 4.1 Genetic diversity analysis of strains

Currently, numerous articles explore the genetic diversity of edible fungi, such as Agaricus blazei (Wan et al., 2021), Pleurotus eryngii (Lin et al., 2023), Lentinula edodes (Wu et al., 2023), and Agaricus bisporus (Gu et al., 2013). These studies employ SSR, ISSR and other molecular marker technologies to cluster strains. In contrast, our study utilizes resequencing technology to analyze individual differences between strains at the genomic level. Our findings demonstrate that resequencing technology is highly effective in analyzing the genetic diversity of strains.

In our study, significant differences in genetic variation between different strains were observed. Through sequencing analysis, we identified substantial variations in genetic makeup among breeds. Notably, strain Or020 exhibited significantly fewer SNPs and InDels compared to other strains, followed by strains Or004 and Or034. The presence of fewer mutation sites suggests more conserved sequences in the evolutionary process, indicating higher genetic stability. These strains may be better suited as primary cultivars. Simultaneously, it is noteworthy that among the detected SNPs, variations in all transmutation types were almost twice as many as in transversion types. This variation predominantly occurred in the exon region (53%) and intronic region (16%), encompassing crucial information for protein synthesis and covering most functional variations related to individual phenotypes. Increased variation in these two regions may resulted in greater phenotypic differences between strains, warranting further investigation into this variation. Regarding the detected InDel variants, the number of deletions and insertions was similar. Mutations in the coding region may lead to changes in protein structure and function, thereby influencing the properties of *H. radicata*. The SNP data were utilized for a group genetic structure analysis dividing the test strains into two subgroups (Figure 3B).

The blue-marked subgroups primarily consisted of strains from Fujian and Shandong, including the main Shandong variety Or034. The number of mutations detected in these subgroups was slightly higher than in the other population, suggesting a higher degree of variability in evolution. Strains with greater variability may hold greater potential for molecular breeding. Conversely, the red-marked subgroups mainly comprised strains from inland areas such as Sichuan and Hunan. The number of variations detected in these strains was slightly lower, indicating a more conservative evolution and stable inheritance of desirable traits. In future breeding efforts, we propose crossbreeding strains from the two subpopulations to obtain heterosis strains, playing a significant role in future production. Additionally, it is essential to collect more varieties for domestication, and expand the germplasm bank of *H. radicata*.

### 4.2 Diversity analysis of fruiting body characters

A comprehensive statistical analysis of yields and fruiting body characteristics across all strains revealed significant differences between the two subgroups in multiple aspects. Thile there is more similarity among strains within the same subgroup, notable differences exist between the two subgroups. Subgroup II demonstrated significant advantages in terms of mycelial growth rate and fruiting body characteristics. However, there was no significant difference in yield traits between the two subpopulations, possibly attributed to greater strain yield variation within subpopulations. In subsequent studies, we recommend hybridizing strains from the two subgroups to select superior strains.

Although the fruiting body characteristics exhibited remarkable quality, the average yield was slightly lower. The comprehensive analysis of cultivation data and biological results demonstrated good consistency, indicating that the selected traits in this study effectively represent the differences and commonalities among strains. These distinctions and similarities are primarily derived from the genetic material of the strains. The study revealed genetic differences among strains but commonalities in yield and fruiting body traits within the same subpopulation. These strains share a common background, potentially representing different strains of the same variety even the same variety. Apart from genetic material, these phenotypic differences and commonalities may also be linked to gene expression and metabolic pathways, which will be the focal point of our future research in analyzing the association between genotype and phenotype.

Furthermore, we observed that strain Or019 exhibits characteristics such as fast mycelial growth, high yield and good fruiting body traits, suggesting its potential as a major cultivar. Similar studies conducted by Lin et al. (2023) on *Pleurotus eryngii* have shown a strong correlation between SNP markers and fruiting body traits, aligning with our findings. In upcoming research, we plan to conduct multi-year cultivation experiments across various regions to investigate the stability of strain yield and fruiting body characteristics. We aim to collect more trait indicators through phenotypic analysis and develop molecular markers for association analysis using the detected variation sites. This will provide essential technical support for variety identification, trait prediction and crossbreeding. Additionally, testing strains’ tolerance to biological and abiotic stresses, screening dominant strains, and optimizing cultivation techniques are envisioned to enhance *H. radicata* quality and yield.

## 5 Conclusion

In this study, we conducted whole-genome re-sequencing on 18 strains of *H. radicata* sourced from different regions in China. The integration of bioinformatics analysis with population genetics allowed us to extract quantitative and positional information about SNPs and InDels. Additionally, the mycelial growth rate, yield, and fruiting body characteristics of the strains were collectively considered to explore commonalities and differences within the population. The 18 materials were categorized into two subgroups, each demonstrating its own set of advantages. Notably, strains within the same subgroup exhibited consistency in both yield and fruiting body characteristics.

For future endeavors, strains from each of the two subgroups can be chosen as parents and crossbred according to breeding objectives, aiming to obtain strains with excellent traits from both subgroups. The molecular markers we identified stand as valuable tools for genetic diversity analysis, gene localization of desirable traits, and the selection of high-quality germplasm in *H. radicata.*

## Data availability statement

The original contributions presented in the study are included in the article/supplementary material, further inquiries can be directed to the corresponding author.

## Author contributions

LC: Conceptualization, Formal analysis, Investigation, Methodology, Writing – original draft. DY: Investigation, Methodology, Resources, Writing – review & editing. QZ: Data curation, Formal analysis, Methodology, Writing – review & editing. YN: Data curation, Formal analysis, Software, Writing – review & editing. WL: Data curation, Investigation, Project administration, Writing – review & editing. RF: Formal analysis, Software, Supervision, Writing – review & editing. WM: Formal analysis, Project administration, Software, Writing – review & editing. XZ: Funding acquisition, Resources, Validation, Visualization, Writing – review & editing.

## Appendix

Agarose gel electrophoresis of the DNA. M: 1 Kb plus DNA ladder marker; 1–18: 18 strains of *H. radicata.*

**Table:**
